# Insights from the rescue and breeding management of Cuvier’s gazelle (*Gazella cuvieri*) through whole‐genome sequencing

**DOI:** 10.1111/eva.13336

**Published:** 2022-02-22

**Authors:** Marina Alvarez‐Estape, Claudia Fontsere, Aitor Serres‐Armero, Lukas F. K. Kuderna, Pavel Dobrynin, Héla Guidara, Budhan S. Pukazhenthi, Klaus‐Peter Koepfli, Tomas Marques‐Bonet, Eulalia Moreno, Esther Lizano

**Affiliations:** ^1^ Institute of Evolutionary Biology, (UPF‐CSIC) PRBB Barcelona Spain; ^2^ Computer Technologies Laboratory ITMO University St. Petersburg Russian Federation; ^3^ Center for Species Survival National Zoological Park Smithsonian Conservation Biology Institute Front Royal Virginia USA; ^4^ Center for Species Survival National Zoological Park Smithsonian Conservation Biology Institute Washington District of Columbia USA; ^5^ Direction Générale des Forêts Tunis Tunisia; ^6^ Smithsonian‐Mason School of Conservation Front Royal Virginia USA; ^7^ CNAG‐CRG Centre for Genomic Regulation (CRG) Barcelona Institute of Science and Technology (BIST) Barcelona Spain; ^8^ Universitat Autònoma de Barcelona (UAB) Edifici ICTA‐ICP Institut Català de Paleontologia Miquel Crusafont Barcelona Spain; ^9^ Catalan Institution of Research and Advanced Studies (ICREA) Barcelona Spain; ^10^ Dept. Ecología Funcional y Evolutiva Estación Experimental de Zonas Áridas‐CSIC Almería Spain

**Keywords:** captive breeding, Cuvier’s gazelle, genomics, inbreeding, reintroduction

## Abstract

Captive breeding programmes represent the most intensive type of ex situ population management for threatened species. One example is the Cuvier’s gazelle programme that started in 1975 with only four founding individuals, and after more than four decades of management in captivity, a reintroduction effort was undertaken in Tunisia in 2016, to establish a population in an area historically included within its range. Here, we aim to determine the genetic consequences of this reintroduction event by assessing the genetic diversity of the founder stock as well as of their descendants. We present the first whole‐genome sequencing dataset of 30 Cuvier’s gazelles including captive‐bred animals, animals born in Tunisia after a reintroduction and individuals from a genetically unrelated Moroccan population. Our analyses revealed no difference between the founder and the offspring cohorts in genome‐wide heterozygosity and inbreeding levels, and in the amount and length of runs of homozygosity. The captive but unmanaged Moroccan gazelles have the lowest genetic diversity of all genomes analysed. Our findings demonstrate that the Cuvier’s gazelle captive breeding programme can serve as source populations for future reintroductions of this species. We believe that this study can serve as a starting point for global applications of genomics to the conservation plan of this species.

## 1 INTRODUCTION

Human activities leading to habitat loss and fragmentation are one of the main threats to biodiversity (Lindenmayer & Fischer, 2013; Ryser et al., 2019; Tee et al., 2018) and, together with other activities such as poaching, can result in isolation of populations, sharp declines in population size and eventually a population bottleneck. This situation could then trigger an extinction vortex. When a small number of animals are left for breeding, genetic drift becomes stronger and inbreeding increases which can reduce fitness, a process known as inbreeding depression (Blomqvist et al., 2010; Keller & Waller, 2002).

In order to preserve biodiversity and prevent the extinction of endangered species, conservation programmes may intend to establish stable captive breeding populations to provide a demographic and genetic reservoir for wild populations. One of the major long‐term goals of captive breeding is the retention of genetic variation to enable future reintroduction efforts (Robert, 2009; Seddon et al., 2007). These events are usually high‐risk endeavours, and although some authors have reported successes (Landa et al., 2017; Linklater et al., 2012) particularly for small populations (Van Houtan et al., 2009), others have not (Fischer & Lindenmayer, 2000; Germano & Bishop, 2009). Several factors have been identified to explain past failures including infections (Northover et al., 2018; Robinson et al., 1998), predation (Grey‐Ross et al., 2009; Moseby et al., 2011), habitat unsuitability (Cook et al., 2010; Sheean et al., 2012) and the use of captive‐bred animals as founders (Bremner‐Harrison et al., 2004; Robert, 2009). In this latter scenario, loss of genetic diversity and inbreeding can occur during captive management and therefore jeopardize the ability of animals to survive in the wild.

Genetic diversity is central to conservation efforts, and it is thus important to consider to what extent captive breeding programmes can maintain it (Coates et al., 2018; Frandsen et al., 2020; Van Dyke, 2008). Traditionally, one of the most relevant tools for the genetic management in captivity of rare and endangered species has been pedigree analysis. Pedigrees enable the retrieval of relevant genetic information such as the level of inbreeding, whose minimization is a primary short‐term goal in such programmes. The inbreeding coefficient (F) indicates the probability that two alleles at a randomly chosen locus are identical by descent (IBD) (Wright, 1922). While there are methods to estimate F from pedigree data (F_PED_) to perform demographic and genetic analysis in captive populations (Ballou et al., 2010; ISIS, 2005; Lacy et al., 2012), variations in Mendelian sampling (Hill & Weir, 2011) and pedigree accuracy might result in inaccurate estimates. Moreover, applying molecular genetic techniques to conservation genetics suggests that they yield more accurate estimates of kinship and the degree of inbreeding (Alemu et al., 2021; Galla et al., 2020). A recent study by Kardos et al. (2018) demonstrated that inbreeding is better measured with whole‐genome data than by F_PED_, for instance by calculating realized inbreeding coefficient (F_RoH_) based on Runs of Homozygosity (RoH). RoH represent tracts of the genome that are homozygous due to the inheritance of equal haplotypes from a common ancestor (Ceballos et al., 2018) and can give insights into population history and consanguinity (McQuillan et al., 2008; Prado‐Martinez et al., 2013; Saremi et al., 2019; Xue et al., 2015). Therefore, F_ROH_ can be used as an alternative to F_PED_ when affordable. However, it has not been until the recent decrease in sequencing costs (Goodwin et al., 2016) and increased availability of genome assemblies for nonmodel species (Farré et al., 2019; Humble et al., 2020) that conservation studies have started to use whole‐genome data to study endangered species, a subfield called conservation genomics (Ellegren, 2014; Feng et al., 2019; Gooley et al., 2020; Saremi et al., 2019; Xue et al., 2015).

Cuvier's gazelle (*Gazella cuvieri*, Ogilby 1841) is a medium‐sized antelope species endemic to the mountain and hill ranges of the Maghreb (northwest Africa), with neighbouring ranges in Morocco, Algeria and Tunisia, where it inhabited the Tunisian Dorsal. Its historical range once spanned from the Mediterranean and Atlantic coast to the northern border of the Sahara (El Alami, 2018; Lavauden, 1924). However, the species has declined dramatically since the 1950s (Beudels‐Jamar et al., 2006) due to excessive hunting, anthropogenic barriers, feeding competition with domestic livestock and habitat degradation (Attum & Mahmoud, 2012; Beudels‐Jamar et al., 2006; Grettenberger & Newby, 1990; Herrera‐Sánchez et al., 2020). Today, only a few small and isolated populations remain (Aulagnier et al., 2015; Beudels‐Jamar et al., 2006; Gil‐Sánchez et al., 2017; IUCN, 2018). The IUCN Red List of Threatened Species classified the Cuvier's gazelle as Endangered between 1986 and 2016. Currently, it is globally classified as Vulnerable (IUCN, 2016), but at a national level, in Morocco and Tunisia this gazelle species is considered Endangered, and it is a Threatened taxon in Algeria (IUCN, 2018). This species is protected under the Convention on International Trade in Endangered Species (CITES) Annex I, and the Convention on Migratory Species (CMS) Annex I.

A captive population of Cuvier’s gazelle was established in 1975 at the La Hoya Experimental Field Station (Almería, Spain) from four founders: 1 male and 3 females (Moreno & Espeso, 2008). The species is managed under a European Endangered Species Programme (EEP), an intensive type of population management for threatened species in European zoological institutions involved in wildlife conservation. The total EEP captive population (for the last 5–10 years) consists of about 160–170 individuals distributed in seven zoological institutions (Espeso & Moreno, 2019). In the population at La Hoya Experimental Field Station, no inbreeding depression has been reported in previous studies (Ibáñez et al., 2011, 2013, 2014; Moreno et al., 2015). Breeding management and husbandry practices in La Hoya Field Station are described elsewhere (Moreno & Espeso, 2008). In October 2016, individuals from the Cuvier’s gazelle EEP were used as founders for a reintroduction project at Jebel Serj National Park in Tunisia (Moreno et al., 2020). Jebel Serj National Park is part of the Tunisian Dorsal, a mountainous chain running from southwest (close to the Algerian border) to northeast (close to the eastern coastal line 16 miles from Tunis) Tunisia. Cuvier’s gazelle inhabited this area up to the 1960s (IUCN, 2018; Moreno et al., 2018). The project used soft‐release techniques where the animals were released into acclimatization pens upon arrival for 3 years. During this 3‐year period (2017–2019), the reintroduced population was monitored. After the arrival in 2016, all females were distributed in breeding herds and only one adult male was included in each breeding herd (*N* = 5). The remaining males were housed in individual enclosures for mating in the following breeding seasons (further details in Moreno et al., 2020). Despite the small number of captive Cuvier’s gazelles used as founder stock (*N* = 43, 31 females and 12 males), the population increased more than expected as indicated by the number of females giving birth, the number of offspring born and those surviving to 30 days from the first (2017) to the third (2019) breeding season (Moreno et al., 2020). The same tendency was detected for the offspring recruitment rate at the population level and for population size. Given these promising results following reintroduction, Moreno et al. (2020) suggested that genetic diversity in captive Cuvier’s gazelles might be higher than what F_PED_ revealed.

In the present study, our objectives are to (i) evaluate the impact of a Cuvier's gazelle reintroduction event on the genomic diversity of their descendants, (ii) estimate and compare inbreeding levels using both genomic and pedigree data and (iii) determine the genetic differences between gazelles from two populations: one managed and one unmanaged. To accomplish these objectives, we have sequenced the genomes of the first and second cohort individuals born after the reintroduction in Tunisia and compared these to the genomes of the reintroduction founders as well as to an unrelated captive but unmanaged population.

## 2 MATERIALS AND METHODS

### 2.1 Study dataset

We included samples from two different Cuvier’s gazelle populations. One population is located in Tunisia (Jebel Serj National Park) as part of a reintroduction project initiated in October 2016 from captive‐bred founders (Moreno et al., 2020). The second population is in Morocco (MOR) and consists of about 60–70 individuals living in a private fenced land (3000 ha) in the Maâmora forest.

**FIGURE 1:**
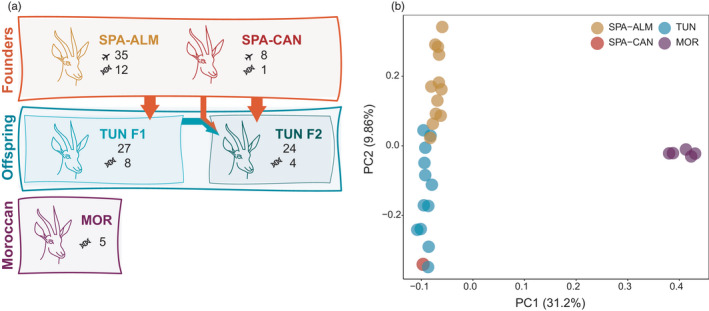
(a) Description of the dataset used in this study. Founder gazelles belong to two different captive populations, the SPA‐ALM from Estación Experimental de Zonas Áridas and the SPA‐CAN from Oasis Park Fuerteventura zoo. Founder gazelles gave rise to the first cohort born in Tunisia (F1). The second cohort (F2) originated from both the mating between only Founder gazelles or between a Founder gazelle and a female from F1. The Moroccan population is a semi‐captive but unmanaged and unrelated population of Cuvier’s gazelles. The airplane icon indicates the number of gazelles used as founder stock in the reintroduction to Tunisia and the DNA icon indicates the number of samples included in our dataset. Numbers without an icon in the offspring indicate the total number of gazelles born in each season. (b) Principal component analysis (PCA) of all gazelles sequenced in this study. SPA‐ALM gazelle in yellow, SPA‐CAN gazelles in red, TUN in blue and MOR in purple

The founder stock of the reintroduced population in Tunisia (*N* = 43, 31 adult females and 12 adult males) originated from two captive populations in Spain (Figure 1a): 35 from the Estación Experimental de Zonas Áridas [(EEZA‐CSIC), Almería, Spain; designated in this study as SPA‐ALM] and eight from the Oasis Park Fuerteventura zoo (Canarias, Spain; designated as SPA‐CAN). Although both founder populations originate from the same four individuals from La Hoya in Almería (Spain, SPA‐ALM) (Espeso & Moreno, 2019), they have been separated since 2006, when 7 gazelles from La Hoya were taken into Oasis Park Fuerteventura in Canarias (Spain, SPA‐CAN) to establish a new captive population (Espeso & Moreno, 2019). Upon arrival, all reintroduced females were distributed among five enclosures forming five breeding groups, with one mating male included in each group at the same time. The remaining seven males were housed in individual enclosures for mating in the following breeding seasons. As a rule, in the first breeding season, males from the SPA‐CAN were included in breeding herds composed solely of females from SPA‐ALM. Similarly, females from SPA‐CAN mated solely with males from SPA‐ALM (additional details in Moreno et al., 2020). For the second breeding season, all males born in the breeding groups were moved to a bachelor enclosure to avoid mating with either their mothers or sisters. However, offspring females were kept in their native breeding enclosure to allow mating with the new adult male selected as breeder from those kept in individual enclosures (see more details for management of breeding groups in Moreno et al., 2020). Female Cuvier's gazelles are fertile from nine months onwards (Moreno & Espeso, 2008); therefore, both founder female gazelles and F1 female gazelles can be progenitors of gazelles born in the second breeding season (F2).

All reintroduced gazelles were ear‐tagged for individual identification. In spring 2017 and spring 2018, the first and the second cohort (F1 and F2) of Cuvier's gazelle were born in Tunisia (*N* = 27 and *N* = 24 calves, respectively, hereafter named Offspring). To facilitate rewilding and to minimize risk of death (stress, infections, abandonment by mother, etc.), calves were not tagged (see (Moreno et al., 2020) for more details). Therefore, from 2017 onward all descendants remained unidentified.

In contrast, the MOR population had no human intervention in mating decisions and although it is kept in a fenced area, we consider it to be an unmanaged but captive population. These Moroccan individuals were unrelated to those reintroduced and consequently to those animals born in Tunisia. This population was founded in 1997 with seven founders (5 females and 2 males, plus another male some years later) from the *Jardin Zoologique de Rabat* (Morocco).

We obtained whole blood samples and sequenced a total of 30 individuals from these two populations (Table S1). Specifically, a total of 13 founders (Founder group, 10 females and 3 males): 12 samples from SPA‐ALM and 1 sample from SPA‐CAN; 12 offspring (Offspring group, 9 females and 3 males): eight born in 2017 (F1) and four born in 2018 (F2); and 5 Moroccan gazelles (Moroccan group, five females) (Figure 1a). Individuals in this study are limited to those gazelles, which freely came into a trapping net to ensure a quick and safe capture for blood sampling. Animal manipulations were performed in accordance with the Spanish Regulation for Animals in Research, RD53/2013, which conforms to European Union Regulation 2010/63/UE on the protection of animals used for scientific purposes.

### 2.2 DNA extraction, library preparation and sequencing

We extracted genomic DNA from whole blood samples using either the Qiagen DNeasy Blood and Tissue Kit (Qiagen), the MagAttract HMW Kit (Qiagen) or the GEN‐IAL All Tissue DNA kit (GEN‐IAL) following manufacturer's protocols (Table S1). Then, we prepared Illumina libraries following the BEST protocol (Carøe et al., 2018), with minor modifications. Briefly, we sheared a total of 200 ng of DNA in 35 µl of lowTE with a Covaris S2 ultrasonicator to an insert size of 300 bp. Libraries were sequenced on a NovaSeq 6000 sequencer (Illumina) to an average depth of ~7× (Table S1).

### 2.3 Filtering and variant calling

First, we trimmed the Illumina sequencing adapters and bases with an average quality <20 using Trimmomatic (version 0.36) (Bolger et al., 2014). Raw data quality was assessed with FastQC (version 0.11.7) (Andrews, 2010) before and after trimming. We aligned the sequencing reads to the chromosome‐length dama gazelle (*Nanger dama*) reference assembly (NCBI GenBank accession GCA_019969365.1) using BWA‐MEM (version 0.7.12) (Li & Durbin, 2009). This assembly has a total contig/scaffold length of 2.977/3.008 Gb, with contig N50 = 0.271 Mb, scaffold N50 = 156.273 Mb and a contig/scaffold L90 of 13,723 contigs/21 scaffolds. The two species are estimated to have diverged ~6 million years ago (Bibi, 2013) and differ in their respective karyotypes: Cuvier's gazelle has 2n = 32, 33 (females and males, respectively), while dama gazelle has 2n = 38–40 (O’Brien et al., 2006). The dama gazelle used for the reference assembly has 2n = 38. While some chromosomal rearrangements might have occurred since the two species split, reference assemblies of different yet evolutionary close species can be used for genotyping (Galla et al., 2019).

To annotate read groups (AddOrReplaceReadGroups) to the corresponding libraries as well as to remove duplicates (MarkDuplicates), we used the Picard software suite (version 1.95) (Broad Institute, 2019). Then, we filtered the resulting BAM files to keep only mapped reads and primary alignments. Next, GATK HaplotypeCaller (version 4.1.4.0) (DePristo et al., 2011; McKenna et al., 2010) was used to call genotypes in each sample independently and GenotypeGVCFs was used for joint genotyping. Furthermore, we filtered the resulting vcf file using GATK SelectVariants ‐‐select‐type‐to‐include (version 4.1.4.0) to keep only variable single nucleotide positions. To include only sites that were covered by 3 to 30 reads and that had a minimum quality of 30, we used VCFtools (version 0.1.12b) (Danecek et al., 2011). We also removed sites that could not be genotyped in more than 20% of the samples and those that had a minor allele frequency lower than 0.02. To further filter the data, we kept only those scaffolds larger than 45Mb (87.23% of the assembly). Also, to remove sex chromosomes we filtered out regions by (i) their depth of coverage and (ii) their alignment to the cattle X‐chromosome (alignment data from Dobrynin et al. (2021)). Specifically, we used MOSDEPTH (version 0.2.6) (Pedersen & Quinlan, 2018) to analyse the coverage in windows. We removed regions in HiC_scaffold_19 (it aligns to the X‐chromosome) that showed more than one standard deviation from the average genome coverage and two standard deviations in the remaining scaffolds. After applying these filters, 73.54% of the assembly remained accessible for the analysis, the callable genome. A final VCF file with a total of 10,197,401 variants was used for all analyses unless specified otherwise.

### 2.4 PCA and admixture

To study population substructure, we used the smartpca utility from the EIGENSOFT (version 7.2.1) software package (Patterson et al., 2006) and a custom R script (version 4.0.1) to plot the results. We performed PCAs using two different sets of samples: the complete set of 30 gazelles and only 4 gazelles, using only one individual from each group of gazelles (SPA‐ALM, SPA‐CAN, Offspring and MOR). We further filtered the VCF to eliminate positions that had missing data in any of the individuals and converted it to PLINK format using VCFtools (version 0.1.12b) (Danecek et al., 2011). This file was used as an input for the ADMIXTURE program (version 1.23) (Alexander & Lange, 2011) to infer population structure, and we used R and RStudio (RStudio Team, 2020) to plot the results. We used VCFTools (version 0.1.12b) (Danecek et al., 2011) to calculate Fst statistics between the different groups of gazelles although the number of gazelles per group is different.

### 2.5 Heterozygosity and RoH

We calculated the number of heterozygous positions in the callable region for each scaffold to retrieve the genome‐wide heterozygosity of each gazelle. Because the samples have an average coverage of ~7×, we used ANGSD (Korneliussen et al., 2014) to estimate genome‐wide heterozygosity from genotype likelihoods and compared it to the estimates from the genotype calling using GATK (DePristo et al., 2011; McKenna et al., 2010). We estimated genome‐wide heterozygosity following filtering for callability (‐sites), using allele frequencies (‐doSaf 1), taking genotype likelihoods into account with the SAMtools algorithm (‐GL 1) and specifying several filters: discard bad reads (‐remove_bads1), use reads where the mate can be mapped (‐only_proper_pairs 1), adjust quality for reads with multiple mismatches to the reference (‐C 50), adjust quality scores around insertion/deletions (‐baq 1), minimum mapping and base qualities of 30 (‐minMapQ 30, ‐minQ 30) include sites with a maximum read length of 30 (‐setMaxDepth 30). Genome‐wide heterozygosity was calculated from the output using realSFS from ANGSD (Korneliussen et al., 2014).

To assess the distribution and density of heterozygous variants across the genomes, we estimated the number of heterozygous positions in genomic windows of 150 kbp with a 100 kbp overlap for every sample. Note that the heterozygosity and callability measures of two adjacent windows will be highly interdependent on account of their shared number of bps. Window heterozygosity was then defined to be the ratio between the number of heterozygous positions and the number of callable bps for every window.

We used window heterozygosity to define RoH via a Hidden Markov Model (HMM) segmentation method. A 3‐state HMM with normally distributed emissions was fitted for each sample, where the state with the lowest heterozygous position density corresponded to the RoH segments and the two remaining states captured any other heterozygosity fluctuations occurring in the genome. For that purpose, the HMM emissions and transitions were fitted as follows: (a) prior emission probabilities were defined by partitioning any heterozygosity windows containing <60,000 noncallable bps into three clusters using the skit‐learn v0.21.2 implementation of the unidimensional k‐means algorithm in python 3.5.2 (Garreta & Moncecchi, 2013). The empirical means, variances and weights of each cluster were then used as prior parameters for the HMM Gaussian emissions. Prior transition probabilities were initialized at random. (b) Both emission and transition probabilities between the three HMM states were then optimized using the python pomegranate v0.11.0 package implementation of the Baum‐Welch algorithm (Schreiber, 2017) on the set of window heterozygosity measures described in (a). The resulting sample‐specific transition probability matrix and emission probabilities were used to segment and classify the window heterozygosity measures. On account of our window size definition, the minimum RoH size produced by this method was 150,001 bps and consisted of at least three adjacent, low‐heterozygosity windows. Because we do not know where the chromosomal rearrangements between dama and Cuvier’s gazelle genomes may have occurred and how this would affect RoH discovery, in this study we will be referring to them as putative‐RoH.

For the comparison of genome‐wide heterozygosity between groups, we used a Tukey Honest Significant Difference test (tukey_hsd from the rstatix R package). To test the significance of the difference in the proportion of the genome in RoH between groups, we used a Wilcoxon test (compare_means (method = ‘wilcox.test’, p.adjust.method = ‘BH’) from the ggpubr R package (https://rpkgs.datanovia.com/ggpubr/). For these analyses, we only indicated the significant differences based on the *p*‐adjusted value of each test.

As a comparison method, we used BCFtools/RoH from the BCFTools package (version 1.10.2) (Narasimhan et al., 2016), which also uses a HMM to identify RoH. The correlation between GATK and ANGSD genome‐wide heterozygosity estimates as well as between the proportion of the genome in putative‐RoH calculated by our HMM or BCFTools/RoH was plotted and estimated with R (ggscatter (cor.coef =TRUE, cor.method = ‘pearson’) from the ggpubr R package).

To examine the genomic colocalization of putative‐RoH among the studied gazelles, we calculated Jaccard coefficients using BEDTools (version 2.27.1) (Quinlan & Hall, 2010) and plotted them using R (Heatmap from the ComplexHeatmap R package version 3.12) (Gu et al., 2016). Jaccard coefficients measure the ratio of shared base pairs in RoH between pairs of gazelles, to the total number of base pairs in the two individuals minus the shared base pairs.

The site frequency spectrum (SFS) was estimated using ANGSD (Korneliussen et al., 2014) and realSFS using the same filters as for the heterozygosity estimates. Because the number of gazelles varies between groups, we downsampled the Founder and Offspring groups to the same N as the Moroccan gazelles (*N* = 5). For this, we generated three random subsets of gazelles for each of these two groups excluding the SPA‐CAN gazelle from any of the Founder subsets.

### 2.6 Inbreeding

Within the EEP population, the inbreeding coefficient (F_PED_) was calculated from the studbook pedigree including all the gazelles in the breeding programme (Espeso & Moreno, 2019) using PMx software (Lacy et al., 2012). The studbook data assume that the initial founders of the captive population of the breeding programme were unrelated and noninbred, which may not be the case. However, considering information published by Valverde (2003) with details of their several capture locations, assumption of unrelated founders seems to be reasonably safe. Although we tried to include samples from those founders in Almería in our study using skin specimens from the scientific collection at the Estación Experimental de Zonas Áridas, skins were chemically treated for preservation such a way that made DNA retrieval impossible. To compare whether the inbreeding coefficient obtained with PMx correlated with our genomic data, we estimated F_HOM_ and F_RoH_ two genomic inbreeding coefficients. F_HOM_ was calculated using VCFTools ‐‐het (version 0.1.12b) (Danecek et al., 2011). It can take negative values and is based on the observed and expected autosomal homozygous genotype counts (Wright, 1948). Since RoH can also be used to study genomic inbreeding, we calculated F_ROH_ (McQuillan et al., 2008) using both the HMM results (F_ROH1_) and the BCFTools/RoH estimates (F_ROH2_). F_ROH_ is the ratio between the length of the genome inside RoH and the total length of the autosomes. Spearman's rho coefficients for all possible pairs (rcorr (type = ‘spearman’) from the Hmisc R package; https://hbiostat.org/R/Hmisc/) were calculated to test the correlation between the different inbreeding estimates. Since we only have F_PED_ estimates for 15 gazelles, its correlation with the other inbreeding estimates only considers these 15 individuals. On the other hand, for the remaining genomic inbreeding estimates the correlation was estimated using all the study gazelles (*N* = 30). We used the Wilcoxon test (compare means (method = ‘wilcox.test’, p.adjust.method = ‘BH’) from the ggpubr R package) to study whether there were significant differences between groups in each inbreeding coefficient and we only retained and report the ones that were significant.

### 2.7 Kinship

We used NgsRelateV2 (Hanghøj et al., 2019), which considers the presence of inbreeding, to estimate relatedness among the 30 gazelles from the study. We also studied relatedness using 25 gazelles excluding the Moroccan individuals, so as to remove any potential population structure between the Moroccan population and the other gazelles. The kinship coefficient of ancestry estimated using NgsRelateV2 is the probability that two alleles are IBD. Next, we compared the kinship coefficient of ancestry from genomic data with the expected relationships from the pedigree. We obtained the pedigree kinship coefficient of all pairs of gazelles from the breeding programme using the studbook data (Espeso & Moreno, 2019) with PMx software (Lacy et al., 2012). The correlation between the genomic and the pedigree kinship coefficients was plotted and estimated with R (ggscatter (cor.coef = TRUE, cor.method = ‘pearson’) from the ggpubr R package). Also, for the visualization of the complex pedigree, we built a simplified genealogical tree representing the closest familial relationships of the Founder gazelles (*N* = 13) and two gazelles from the Offspring (the only two for which familial relationships were known) (Figure S1).

## 3 RESULTS

### 3.1 Population differentiation

We evaluated the genomic diversity and population differentiation through different cohorts of a reintroduced Cuvier's gazelle population originated from an initial ex situ captive breeding programme. We generated whole‐genome sequences for a total of 30 individuals to an average depth of coverage of ~7× (4.77×–14.60×) (Table S1), which has been determined to be sufficient for the purposes of our study (Benjelloun et al., 2019). The dataset consisted of 13 Founder individuals, 12 of their offspring born in Tunisia and five representatives from a captive but unmanaged population in Morocco, unrelated to neither the Founders nor their Offspring (Figure 1a). Our sample of 13 Founder individuals (12 from SPA‐ALM and 1 from SPA‐CAN) represent up to 30% of the total founder stock. The 12 Offspring gazelles are from the first and second cohort (F1 and F2) offspring and represent 28% of the total offspring surviving more than 30 days (Moreno et al., 2020).

Principal component analysis separates the Moroccan population from the rest of the gazelles along PC1 (31.2%) (Figure 1b). The second component (PC2 9.86%) explains most of the variation between Founder and Offspring gazelles, with the two founder populations separating at the extremes (SPA‐ALM and SPA‐CAN) and their offspring (TUN) falling between them. PC2 explains a similar fraction of the variance as PC3 and PC4 (Figure S2). Fst values between groups of gazelles indicate the same population stratification obtained with PCA (Weighted Fst_(SPA‐ALM vs. SPA‐CAN)_ = 0.02 and Weighted Fst_(Founders vs. Moroccan)_ = 0.258), even though we only have one individual from SPA‐CAN. To remove any potential bias introduced by having related individuals in the PCA, we performed this analysis including a single gazelle of each group (SPA‐ALM, SPA‐CAN, Offspring/TUN, MOR). This PCA shows a similar clustering of the gazelles as the one including all individuals (Figure S3). The structure analysis of the 30 sequenced gazelles shows that the Moroccan gazelles have a different genetic ancestry compared to the rest (Founders and Offspring), which remains consistent after subsampling the dataset to a similar number of individuals per group (Founders, *N* = 6; Offspring, *N* = 5; and Moroccan, *N* = 5) (Figure S4).

### 3.2 Heterozygosity

**FIGURE 2:**
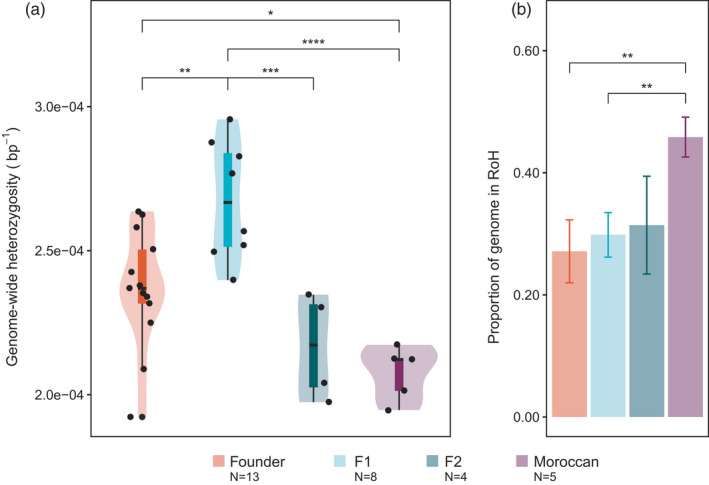
(a) Genome‐wide heterozygosity (bp^−1^) within groups (Founders, *N* = 13; Offspring F1, *N* = 8, Offspring F2, *N* = 4 and Moroccan, *N* = 5). ANOVA (*p* = 5.31 × 10^−5^) and Tukey HSD (*p*.adj_Founder‐F1_ = 6.63 × 10^−03^, *p*.adj_Founder‐Moroccan_ = 3.29 × 10^−02^, *p*.adj_F1‐F2_ = 9.18 × 10^−04^, p.adj_F1‐Moroccan_ = 4.47 × 10^−05^). Boxplots show the median, the 25th and 75th percentiles, Tukey whiskers (median ± 1.5 times interquartile range). (b) Median proportion of the genome in RoH for each group with standard deviation represented as error bars. (Wilcoxon Test, *p*.adj_Founder‐Moroccan_ = 1.4 × 10^−3^, *p*.adj_F1‐Moroccan_ = 1.5 × 10^−3^). *p.adj < 0.05, **p.adj < 0.01, ***p.adj < 0.001, ****p.adj < 0.0001. In (a) and (b), Founder gazelles are coloured in orange, F1 in light blue, F2 in green and Moroccan gazelles in purple

Genome‐wide heterozygosity, measured as heterozygous positions per base pair of the callable genome, is significantly lower in the Moroccan gazelles (median_Moroccan_ = 2.1 × 10^−4^) compared to the individuals born in Tunisia (*ANOVA p* = 2.2 × 10^−2^, *Tukey HSD p*.*adj_Offspring_*_‐_ *_Moroccan_* = 7.57 × 10^−3^). The median genome‐wide heterozygosity in the offspring of the reintroduced gazelles is similar to that of the Founder gazelles (Figure 2a, median_Founders_ = 2.36 × 10^−4^ vs. median_Offspring_ = 2.5 × 10^−4^). When separating the Offspring gazelles into F1 and F2, we observe the highest genome‐wide heterozygosity levels in the first cohort (Figure 2a, median_F1_ = 2.66 × 10^−4^ vs. median_F2_ = 2.17 × 10^−4^), which significantly differ from all the other sampled individuals. We observe that heterozygosity is variable among different scaffolds; Hi‐C_scaffold_10 has the highest heterozygosity levels, while scaffolds 6 and 18 have the lowest (Figure S5). The genome‐wide heterozygosity estimates computed with ANGSD are slightly lower yet consistent with the GATK results. Both estimates show a statistically significant correlation (Figure S6). When analysing the SFS, we find the Founder gazelles to have more fixed unique alleles compared to the other groups (Figure S7).

We have identified a total of 16,371 RoH, with an average number of 521.23 ± 51.96 (Founders), 521.875 ± 37.64 (F1), 580 ± 20.73 (F2) and 620 ± 21.54 (Moroccan) events per gazelle group. Moroccan gazelles have a significantly higher proportion of RoH compared to the other gazelles, while there is no statistical difference between the Founder and the Offspring groups (Figure 2b). Breaking down the Offspring into F1 and F2, we observe that the second cohort displays a slightly higher but nonsignificant genomic RoH proportion. When splitting RoH by size, we observe that the Moroccan gazelles have a significantly larger proportion of RoH for all size categories compared to both Founders and Offspring F1, with more differences in number for longer RoH (>1 and >10 Mb) (Figure S8). The two gazelles with the highest genomic proportion of RoH are G29 and G26 (47%), from the Moroccan population, and the gazelle with the lowest is G05 (21%), a founder from SPA‐ALM (Table S1).

We also analysed RoH with the publicly available software BCFTools/RoH, a different HMM‐based method. The RoH estimated by this method are highly fragmented and therefore shorter, which results in about half the genomic coverage of RoH as our custom HMM method. Nonetheless, both methods have a significant positive correlation (Figure S9).

Runs of Homozygosity are differentially distributed along the Hi‐C scaffolds among individuals and groups of gazelles (Founders, Offspring and Moroccan) (Figure S10A). After a pairwise comparison between all gazelles, we detect that gazelles G15 and G19 have the highest proportion of shared RoH (Jaccard Coefficient = 0.829, Figure S10B). Most of the RoH are shared by a small number of gazelles, and we do not find a population that has a higher amount of RoH shared by most gazelles compared to private RoH (Figure S10C).

### 3.3 Inbreeding and relatedness analyses

**FIGURE 3:**
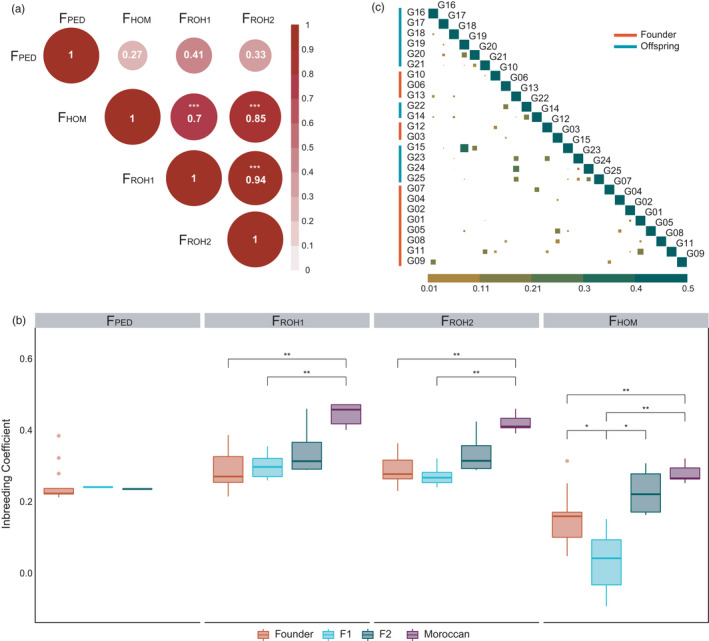
(a) Correlation matrix of inbreeding coefficients. F_PED_ has *N* = 15, and the other estimates have *N* = 30. Colour intensity and circle size are proportional to the correlation coefficients. Values inside circles indicate correlation coefficients (see Table S2), ***p<0.001. (b) Inbreeding levels in each population estimated using the pedigree information (F_PED_, *N* = 15) and genomic information (F_ROH1_, F_ROH2_, F_HOM_, *N* = 30). Wilcoxon test with Benjamini–Hochberg (BH) correction for multiple testing (F_ROH1_: *p*.adj_Founder‐Moroccan_ = 1.8 × 10^−3^, *p*.adj_F1‐Moroccan_ = 4.7 × 10^−3^; F_ROH2_: *p*.adj_Founder‐Moroccan_ = 1.8 × 10^−3^, *p*.adj_F1‐Moroccan_ = 4.7 × 10^−3^, F_HOM_: *p*.adj_Founder‐Moroccan_ = 7 × 10^−3^, *p*.adj_Founder‐F1_ = 9.7 × 10^−2^, *p*.adj_F1‐Moroccan_ = 4.7 × 10^−2^, *p*.adj_F1‐F2_ = 9.3 × 10^−3^). Boxplots show the median, the 25th and 75th percentiles, Tukey whiskers (median ± 1.5 times interquartile range). *p < 0.05, **p < 0.01, ***p < 0.001. Founder gazelles are coloured in orange, F1 in light blue, F2 in green and Moroccan gazelles in purple. (c) Kinship analysis of the Founder and Offspring gazelles. Kinship coefficient (Θ) of one individual with itself or a monozygotic twin equals 0.5. The lower the Θ value, the more distant is the familial relationship. Colour intensity and square size are proportional to the familial relationship estimated by NgsRelateV2

We observe no significant correlation between the inbreeding coefficient based on pedigree data (F_PED_) and any other genomic inbreeding estimates (F_ROH1_, F_ROH2_ and F_HOM_) (Figure 3a). In contrast, all genomic inbreeding coefficients other than F_PED_ have a significant correlation between them (Table S2). All genomic inbreeding estimates indicate that the Moroccan group has the highest inbreeding levels compared to the other groups (Figure 3b and Table S1).

Kinship analysis confirms that Moroccan gazelles are not related to any of the other gazelles but they show high levels of relatedness amongst themselves (Figure S11). Overall, the kinship coefficient obtained with NgsRelateV2 and the expected kinship coefficient from studbook data are positively correlated (Figure S12) although in some cases the observed kinship coefficient is lower than the realized kinship estimated from molecular data (Table S3). The two gazelles in Tunisia with the highest kinship coefficient are G15 and G19 (Offspring F1) (Figure 3c), which also share most of their RoH (Figure S10B). If we focus only on the gazelles from which there is pedigree information (all Founder gazelles and Offspring gazelles: G14, F22; Figure S1, Table S3), we observe that the highest kinship coefficient is for the pair between G01 and G11, which do not seem to be close in the genealogy. Nonetheless, the next higher values belong to parent‐offspring relationships that can be observed in the genealogy from Figure S1 (G14‐G22, G03‐G05, G06‐G22, G07‐G14).

## 4 DISCUSSION

Genomic approaches are becoming increasingly used by captive breeding programmes to analyse the levels of genetic diversity and inbreeding of captive‐bred animals. They can be applied to study the progress of reintroduction programmes on the recovery of an endangered species. To assess the genetic diversity of the cohort born after the reintroduction of 43 Cuvier's gazelles back into the wild, we have sequenced 30 complete genomes, representing the first genomic dataset available from this species. Our study includes a subset of captive‐bred gazelles that were reintroduced into Tunisia as founders, as well as some of their offspring. These gazelles are compared to one another and to an unmanaged Cuvier's gazelle population in Morocco.

Despite the increase of genomic studies on nonmodel species (Genereux et al., 2020), there are still many organisms that lack a reference genome or have never been sequenced, such as Cuvier's gazelle. Consequently, for our analyses, we used a chromosome‐length assembly of a closely related species, the dama gazelle (*Nanger dama*). Recent studies have shown the usefulness of employing assemblies from other species for conservation management (Galla et al., 2019) and population genomic analyses (Prasad et al., 2021).

A Cuvier's gazelle population was reintroduced back into Tunisia in 2016 from captive founders with successful results in terms of fitness traits after their local extinction (Moreno et al., 2020). This suggests that, while in captivity, the animals did not accumulate significant amounts of deleterious alleles that would hinder their recovery when released in nature (Moreno et al., 2020). Our results show that those gazelles born after the reintroduction have similar genome‐wide heterozygosity compared to the Founders (including 12 SPA‐ALM gazelles and a single individual from SPA‐CAN). Furthermore, gazelles from the first cohort in Tunisia (F1) have a significant increase in heterozygosity levels, which is likely the result of a successfully managed breeding programme in the first cohort with minimum coancestry matings, where gazelles from SPA‐ALM of one sex were housed with gazelles from SPA‐CAN of the other sex. This situation may have favoured a different fluctuation of allele frequencies due to random drift in each group. This effect is shown in the second component of the PCA analysis and in the structure analysis (*K* = 3) where both founder populations are clearly separated. Overall, our results suggest that mixing the SPA‐ALM and SPA‐CAN gazelles, which had no gene flow for two generations, has avoided the loss of genetic diversity and even resulted in a slight increase in genome‐wide heterozygosity in the F1 Offspring cohort. Our findings are in line with previous publications stating that genetic diversity in several endangered species can be increased, by including individuals from different populations (Biebach & Keller, 2012; Frankham, 2008; Rick et al., 2019).

We demonstrate that the captive management at La Hoya Field Station as described in Moreno & Espeso (2008) (see also Moreno et al., 2015) is successful in (i) managing genetic diversity at a population level and (ii) producing individuals retaining significant potential for adapting to a new indigenous environment if used as founders in reintroductions.

We observe that the Moroccan gazelles, which belong to an unmanaged but captive population, show different genetic characteristics from the rest of the gazelles included in this study: they display the lowest genome‐wide heterozygosity levels and the highest genomic RoH proportion of any size category, probably due to a lower effective population size compared to the reintroduced Tunisian gazelles (realized effective population size of the gazelles in Almería has been estimated to be 14 by López‐Cortegano et al., 2021). Our findings are in concordance with previous studies where unmanaged captive populations have been shown to display lower genome‐wide heterozygosity and higher levels of inbreeding than managed captive populations (El Alqamy et al., 2012; Gooley et al., 2020; Humble et al., 2020).

Levels of inbreeding play a fundamental part in species survival and the success of reintroductions. Keeping a complete and accurate pedigree of a species in captivity (Ito et al., 2017) or under a conservation programme is often very difficult (Jiménez‐Mena et al., 2016; Putnam & Ivy, 2014). The Cuvier's gazelle studbook (Espeso & Moreno, 2019) is one of the most complete for captive populations (99.5% of pedigree completeness; López‐Cortegano et al., 2021). It contains the recorded familial relationships of the 13 Founder gazelles in our dataset and represents a unique opportunity to compare F_PED_ to other genomic inbreeding estimates, such as F_ROH_. It is known that F_PED_ is based on the expected proportion of the genome that is IBD between two parents, but it does not capture the variation originating from Mendelian sampling and linkage during gamete formation (Hill & Weir, 2011). Also, the studbook of the Cuvier's gazelle conservation programme assumes unrelated and noninbred founders, which might not be the case and can result in underestimated inbreeding estimates (Hogg et al., 2019). We know the exact familial relationships of 15 gazelles, 13 of which belong to the Founder group and 2 of which are Offspring born in Tunisia. The other gazelles from the Offspring group were not identified by ear tags or other marks and therefore could not be recorded in the studbook. Yoshida et al. (2020) compared F_PED_ to other inbreeding estimates and suggested that genomic inbreeding estimates may be more accurate, which is in accordance with our results. The fact that, as expected, we do not find a correlation between the inbreeding coefficient based on F_PED_ and the genomic inbreeding coefficients highlights the value of applying genomics to conservation and captive breeding. F_PED_ can be blind to background inbreeding and inaccurate kinship. Although pedigrees are still a good source of information (Galla et al., 2020; Wang, 2016), genomic approaches can help gain a better understanding of the genomic makeup of the species under study and their levels of inbreeding (Saremi et al., 2019). Sequencing costs have been decreasing since their appearance and have been increasingly applied to the study of endangered species (Genereux et al., 2020). However, the limited funding as well as the high computational resources needed can impair the generalization of such analyses in conservation (Fuentes‐Pardo & Ruzzante, 2017; Shafer et al., 2015). Yet, genomics can be especially useful if pedigree data is lacking, as is the case of unmanaged populations. Moroccan gazelles show the highest levels of inbreeding for all genomic estimates. This is consistent with the low genetic diversity levels and the high proportion of their genome within RoH. The breeding of SPA‐ALM and SPA‐CAN gazelles for the reintroduction of Cuvier's gazelles in Tunisia may be the reason why the inbreeding coefficient in the Offspring group is maintained (F_ROH1_ and F_ROH2_) or even reduced (F_HOM_).

We performed a kinship analysis to further examine the underlying relationship between gazelles and confirmed that the Moroccan gazelles are unrelated to the other gazelles studied. For the offspring group, we hypothesize that G15 and G19 are dizygotic twins from inbred parents because they exhibit the most extreme levels of shared RoH in our dataset, and the coefficient of kinship for this pair is higher than for any other pair of gazelles, even parent‐offspring relationships. From the field records of the reintroduction programme (Moreno et al., 2020), we know that G15 had a twin, but the other gazelle was not identified until now. Interestingly, both gazelles are related to G20, which explains why the three gazelles cluster together in the PCA along the third component. Thus, we acknowledge that the PCA may be influenced by not only population differentiation, but also by familial distances on account of related samples (Rodríguez‐Ramilo et al., 2014). Therefore, we performed a PCA analysis with a single gazelle per group. The kinship coefficient from molecular data detects parent–offspring and half‐sibling relationships that can be confirmed with the pedigree data and the genealogy built from the studbook records. The realized kinship values show a positive correlation with the expected kinship values from the studbook pedigree. Importantly, pedigree estimates assume noninbred and unrelated founder individuals of the captive population, but we cannot totally rule out the possibility of founders being related in this population, although it does not seem to be the case considering information in (Valverde, 2003).

In this study, we showed how genomic tools are useful to assess the genomic composition of individuals that are part of an ex situ conservation project and their offspring. In this particular case, we have assessed the consequences of a reintroduction event after it was carried out. The characterization of the genomic status of the founder gazelles and the first offspring born after the reintroduction sets the grounds for future projects of genetic monitoring of the descendants of this initial release. When possible, future conservation plans could go one step further and use genomics not only to evaluate the success after the reintroduction has already been carried out but also to provide further information for the selection of the best‐fitted animals as founder stock, together with other indicators that may be used to select individual animals for reintroduction. It would have been very interesting to compare the diversity and inbreeding of the founders of the captive population in La Hoya, founded in 1975, to the diversity of the gazelles used as Founders in Tunisia. Sadly, the only remaining samples from the gazelles from the former population were skins chemically treated for preservation in a museum, which made DNA retrieval impossible. Sampling of specimens is advisable for endangered species whenever possible. Blood and skin samples from animals in the La Hoya Field Station are stored in a Genetic Rescue Bank that was started in 2003.

Considerable uncertainty exists regarding the ability to maintain genetic diversity in captive‐bred populations in long‐term management programs and the suitability of these animals for the re‐establishment of populations into the native historical range of a species. Altogether, we have demonstrated that captive‐reared individuals of EEP Cuvier's gazelle were suitable as founders for a reintroduction in Tunisia during 2016, as proven by both animal survival and other fitness traits (Moreno et al., 2020) and by observing no major genetic differences between Founders and Offspring. With this study, we have shown that genomics can aid conservation projects to guide and assess the success of a reintroduction event, particularly by using metrics for inbreeding estimates. We have determined that a captive‐bred gazelle population can be a suitable source for reintroduction into the wild.

## CONFLICT OF INTEREST

The authors declare no conflict of interest.

## Supporting information

Fig S1‐S12

Table S1‐S3

## Data Availability

The data that support the findings of this study are openly available in the European Nucleotide Archive (ENA) at EMBL‐EBI at https://www.ebi.ac.uk/ena/browser/view/PRJEB41985, reference number PRJEB41985.
