# Supplementary material for: Insights from the rescue and breeding management of Cuvier’s gazelle (Gazella cuvieri) through whole‐genome sequencing
Source: Evol Appl. 2022 Feb 22;15(3):351–64. doi: 10.1111/eva.13336 (PMC8965372; doi:10.1111/eva.13336)
Supplement: Supplementary file 1 — Fig S1‐S12 [file EVA-15-351-s001.pdf]

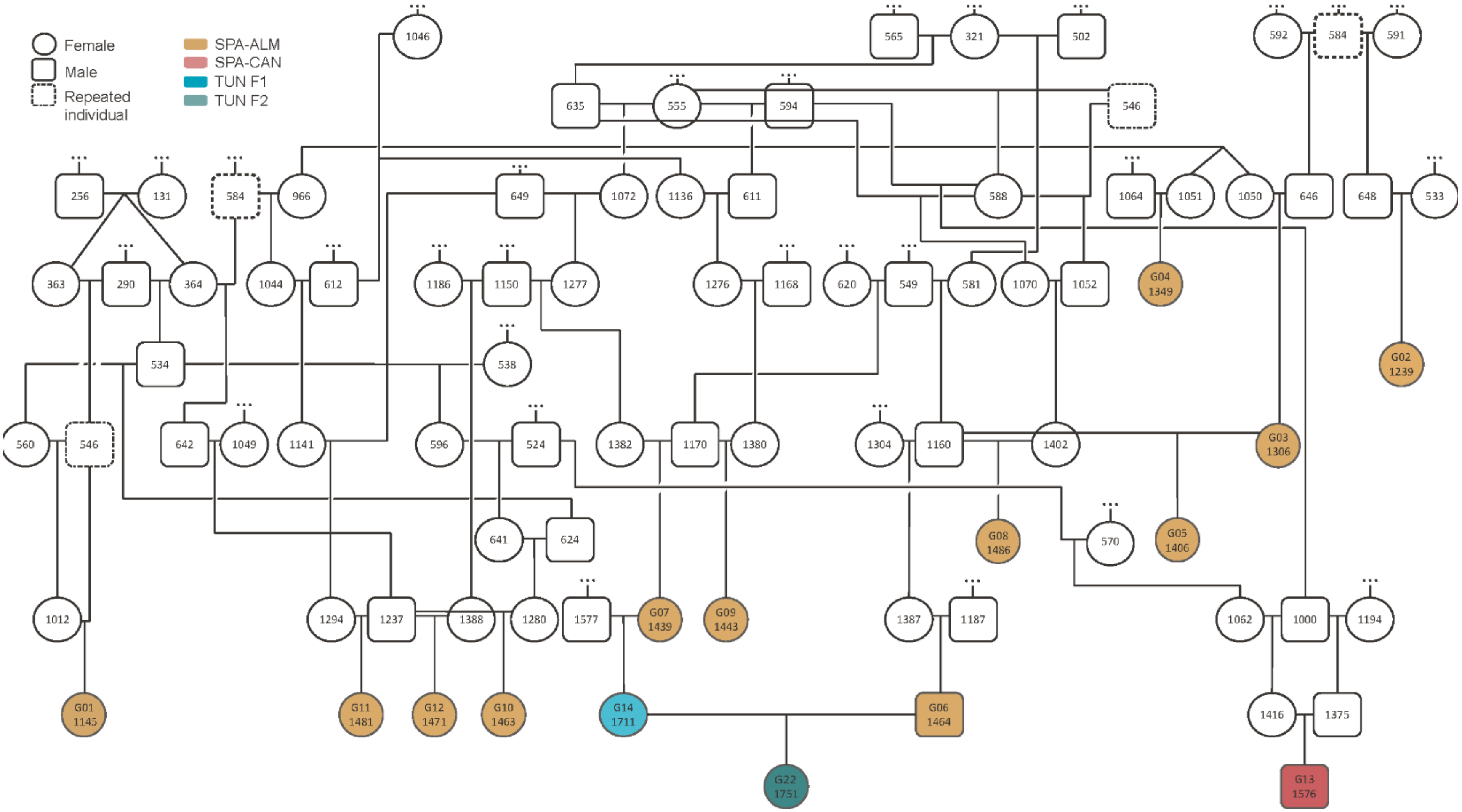


**Figure S1.** Genealogical tree of Cuvier's gazelles based on studbook information (Espeso and Moreno, 2019). The 12 Founder gazelles from SPA-ALM (La Hoya, Almeria, Spain) are colored in yellow, the Founder gazelle from SPA-CAN (Oasys Park Fuerteventura zoo, Fuerteventura, Spain) in red, G14 gazelle that belongs to F1 Offspring cohort born in Tunisia in light blue and G22 gazelle that belongs to F2 Offspring cohort born in Tunisia in green. Pedigree data for the rest of Gazelles born in Tunisia is not available. Three dots on top of some gazelles represent those ancestors not represented in the genealogy to simplify the visualization of the familial relationships of interest in this study. Numbers inside figures are the corresponding studbook IDs for each individual.

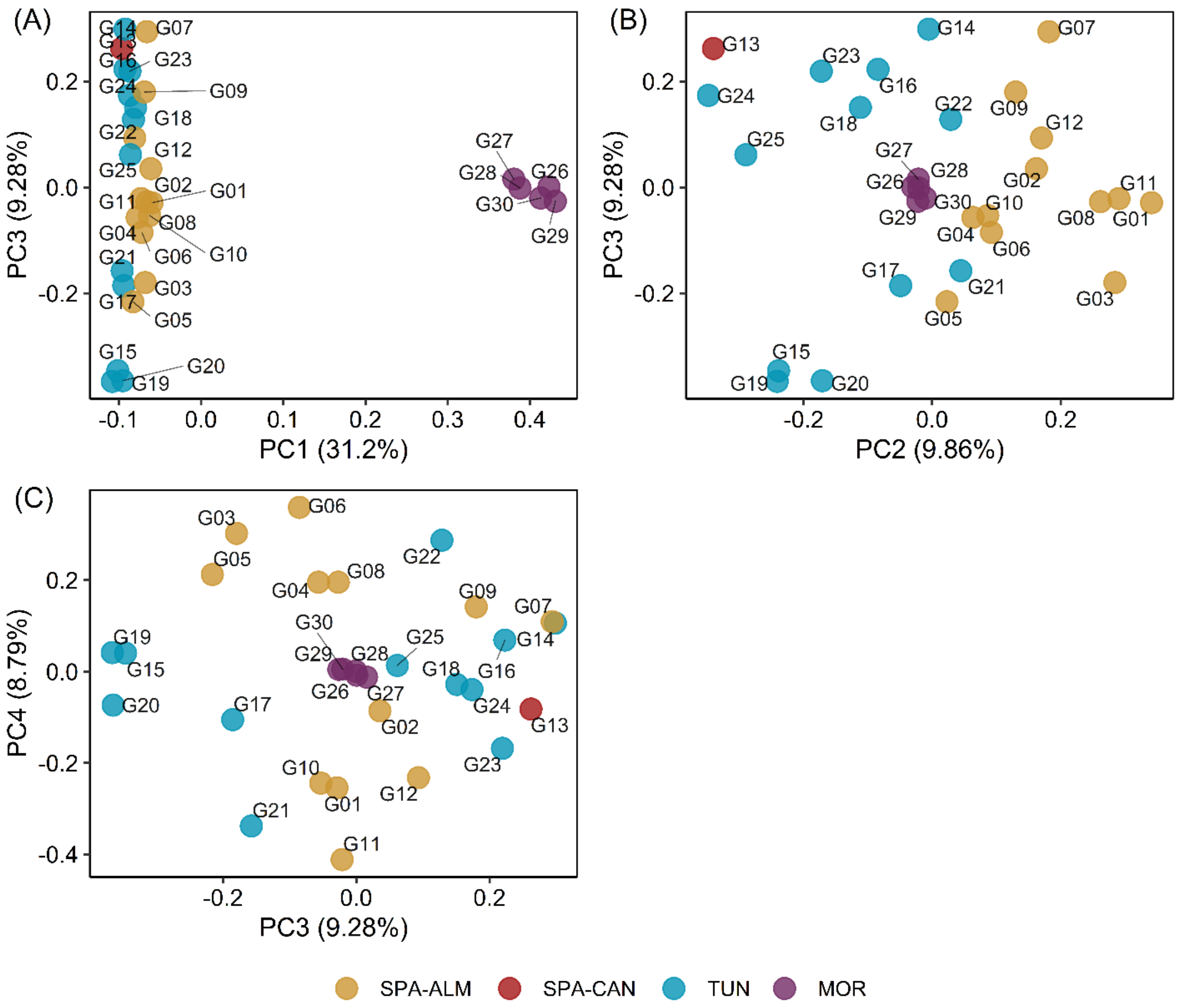


**Figure S2.** PCA of Cuvier's gazelles labelled by their origin and sample name. (**A**) PC1 vs PC3, (**B**) PC2 vs PC3, and (**C**) PC3 vs PC4.

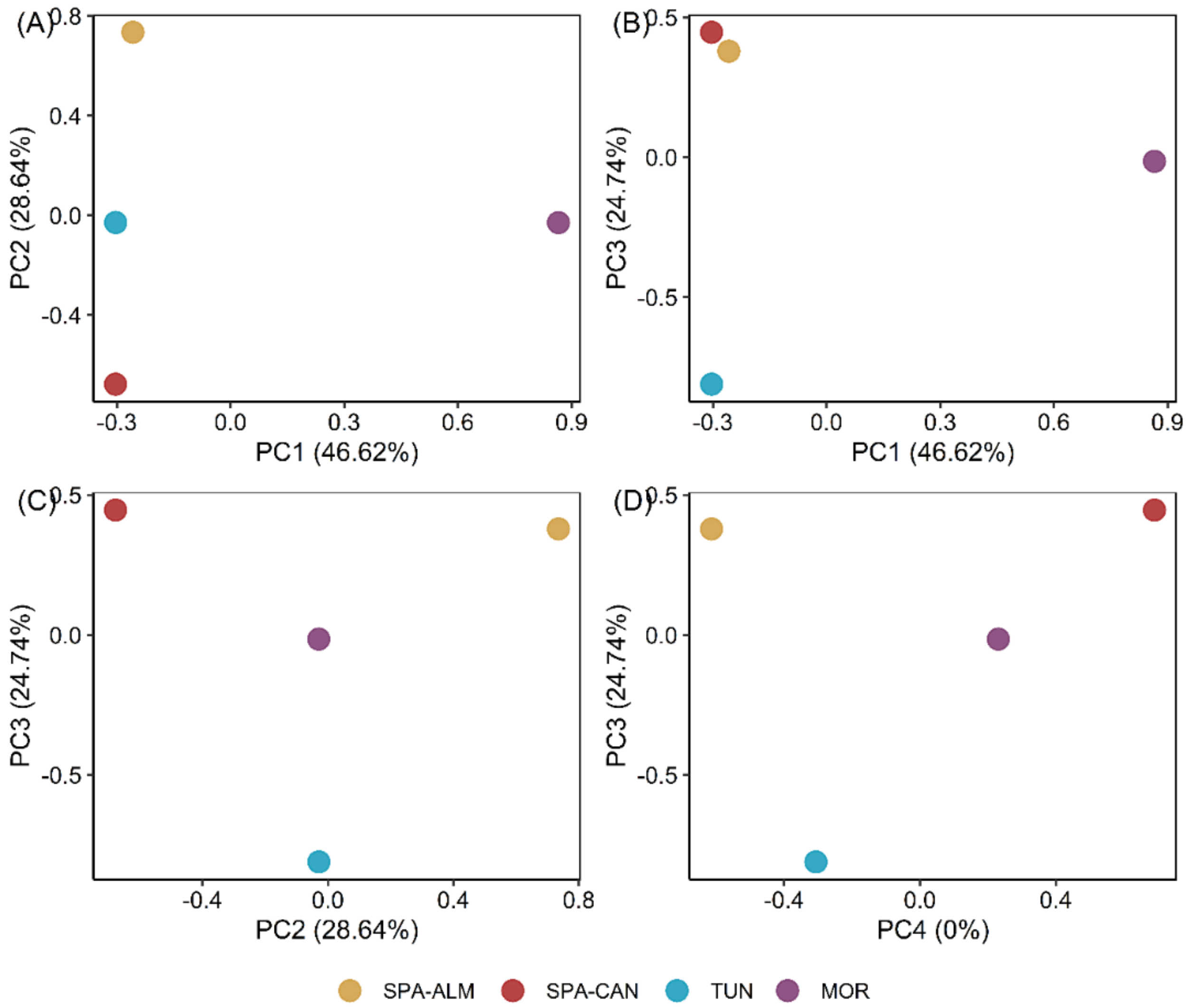


**Figure S3.** PCA computed using 1 gazelle per group (SPA-ALM, SPA-CAN, TUN and MOR gazelles) to remove the effect of having related individuals when computing the PCs. (**A**) PC1 vs PC2, (**B**) PC1 vs PC3, (**C**) PC2 vs PC3, and (**D**) PC3 vs PC4.

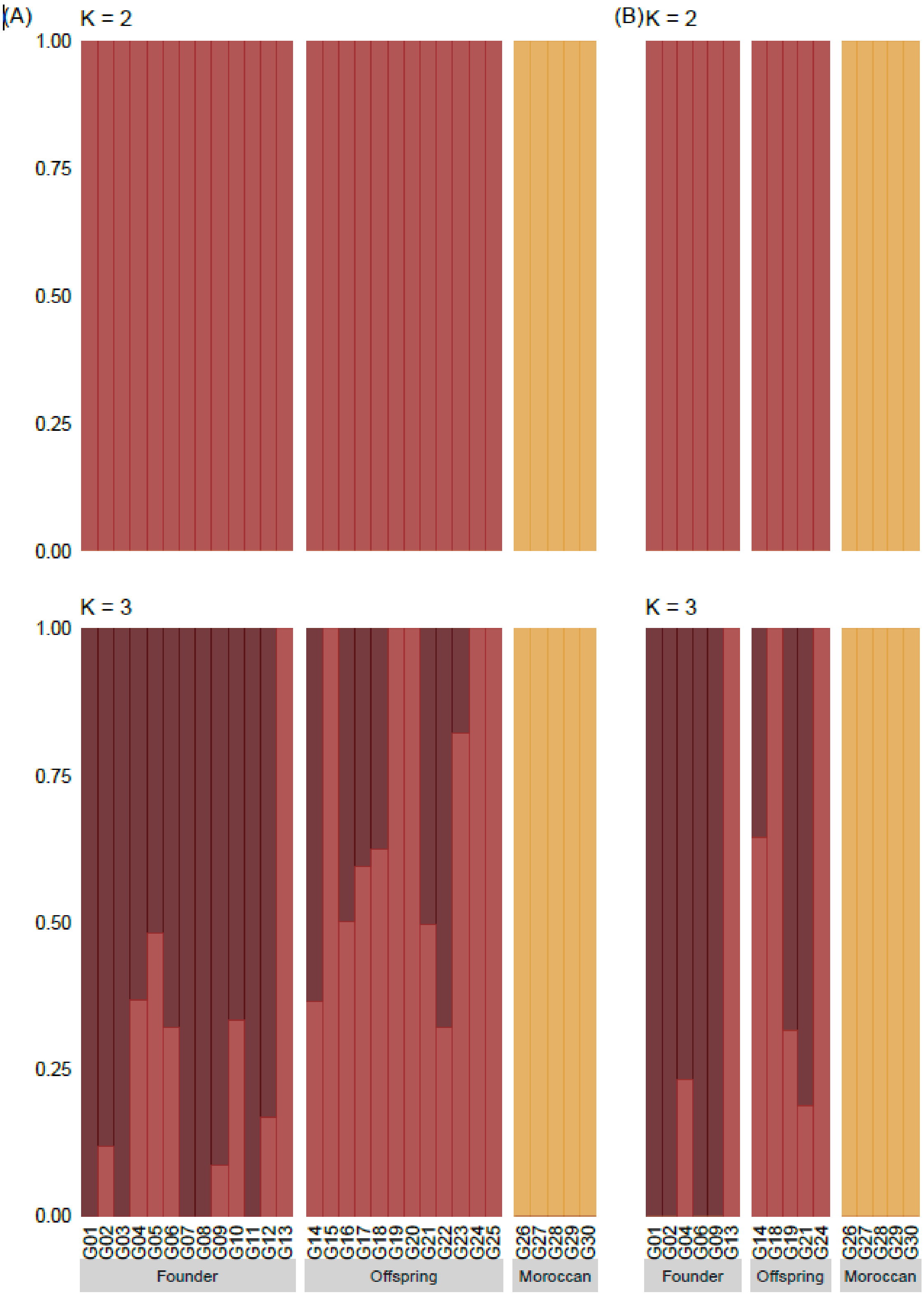


**Figure S4.** ADMIXTURE analysis (**A**) using 30 gazelles and (**B**) a subset of 16 gazelles.

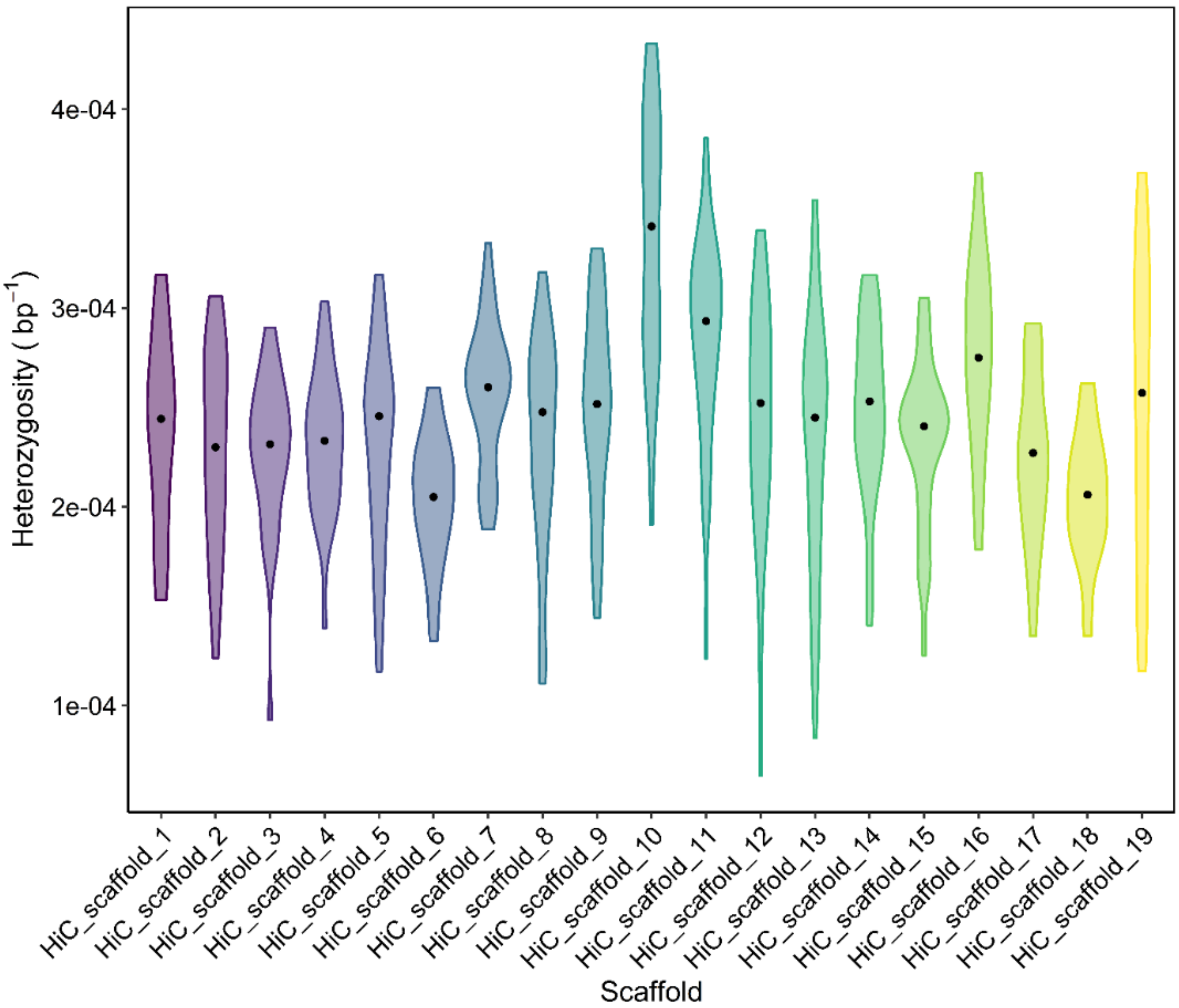


**Figure S5.** Genomic heterozygosity levels by scaffold.

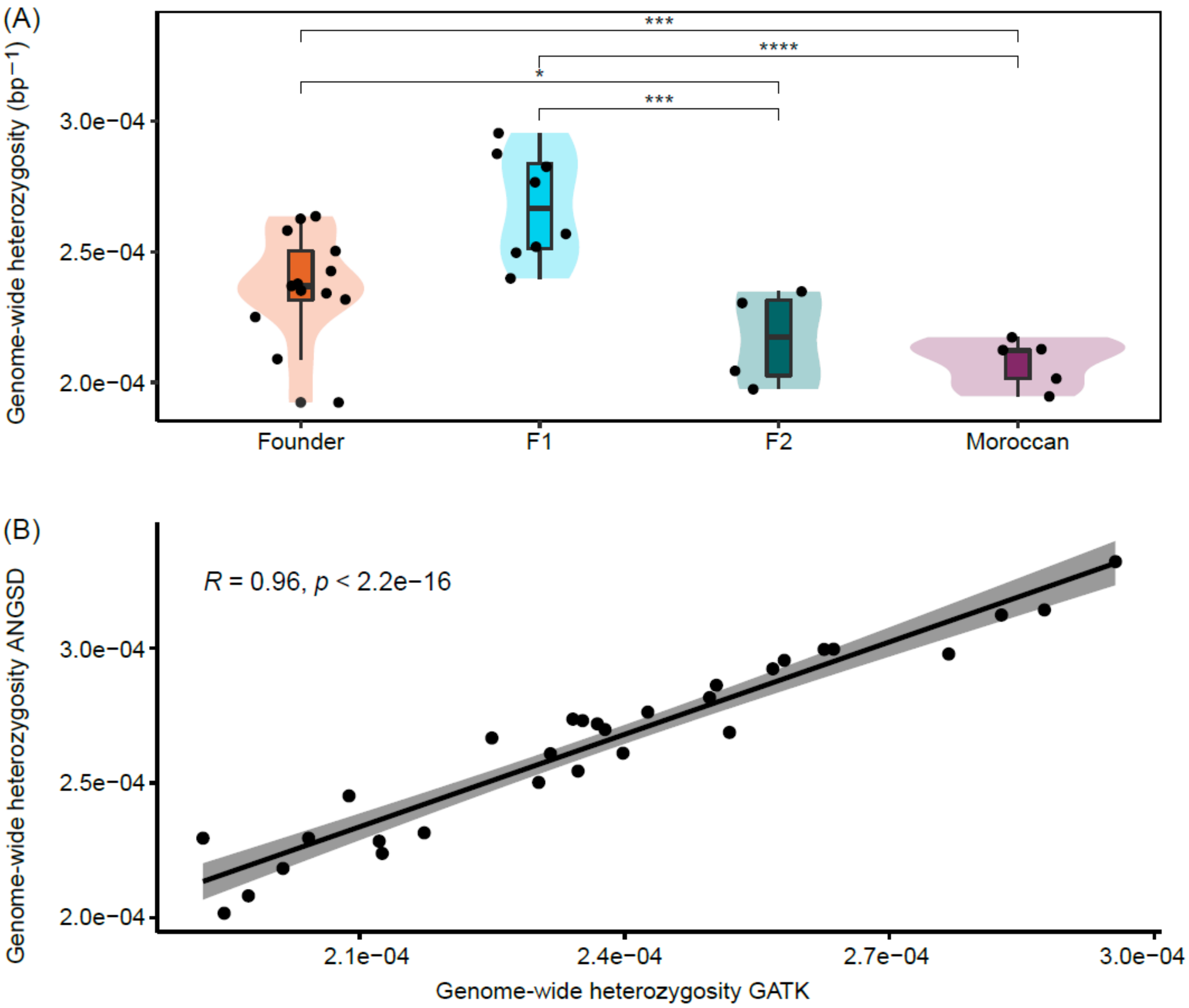


**Figure S6.** (**A**) Genome-wide heterozygosity levels in each group (Founders, N = 13; Offspring, N = 12 and Moroccan, N = 5. Tukey HSD ($p.\mathrm{adj}_{\text{Founder- F2}} = 1.85\times10^{-2}$, $p.\mathrm{adj}_{\text{Founder-Moroccan}} = 2.92\times10^{-4}$, $p.\mathrm{adj}_{\text{F1-F2}} = 3.78\times10^{-4}$, $p.\mathrm{adj}_{\text{F1-Moroccan}} = 5.8\times10^{-6}$)). Boxplots show the median, the 25th and the 75th percentiles, Tukey whiskers (median ± 1.5 times interquartile range). *$p<0.05$, **$p<0.01$, ***$p<0.001$, ****$p<0.0001$. Founder gazelles are colored in orange, Offspring in blue and Moroccan gazelles in purple. (**B**) Pearson correlation between heterozygosity values estimated using hard calls with GATK and using genotype likelihoods with ANGSD.

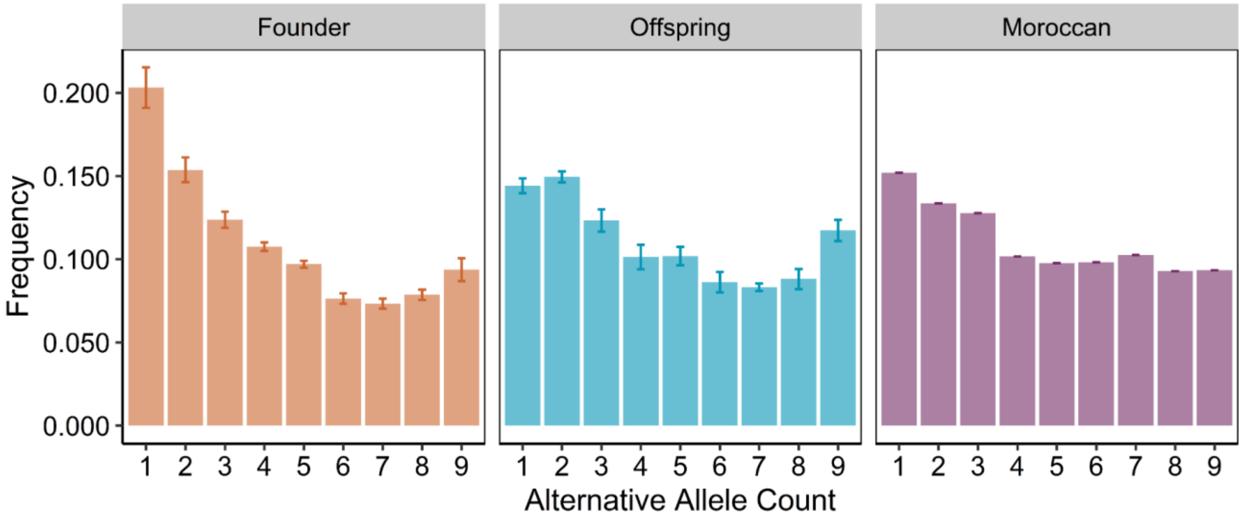

**Figure S7.** Site Frequency Spectrum (SFS) for the 3 groups of gazelles (Founder, Offspring and Moroccan). Error bars represent SD in Founders and Offspring using 3 random subsets of N=5 gazelles. Founder gazelles are colored in orange, Offspring in blue and Moroccan gazelles in purple.

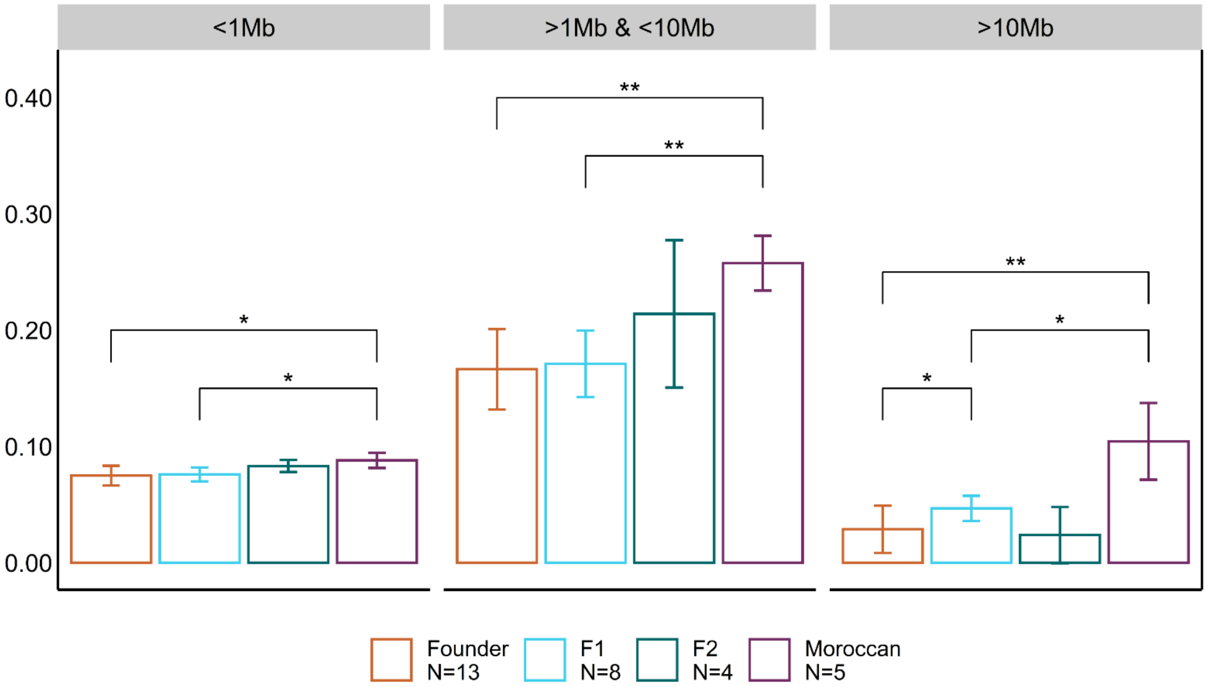


**Figure S8.** Median proportion of the genome in RoH for each group with standard deviation. RoH proportions are analyzed in bin-sizes of <1Mb (Wilcoxon Test, $p.adj_{Founder\text{-}Moroccan} = 2.4\times10^{-2}$, $p.adj_{F1\text{-}Moroccan} = 3.3\times10^{-2}$), 1Mb-10Mb (Wilcoxon Test, $p.adj_{Founder\text{-}Moroccan} = 8.4\times10^{-3}$, $p.adj_{F1\text{-}Moroccan} = 9.3\times10^{-3}$) and >10Mb (Wilcoxon Test, $p.adj_{Founder\text{-}F1} = 3.3\times10^{-2}$, $p.adj_{Founder\text{-}Moroccan} = 8.4\times10^{--3}$, $p.adj_{F1\text{-}Moroccan} = 1.4\times10^{-2}$). Founders are colored in orange, Offspring in blue and Moroccan gazelles in purple.

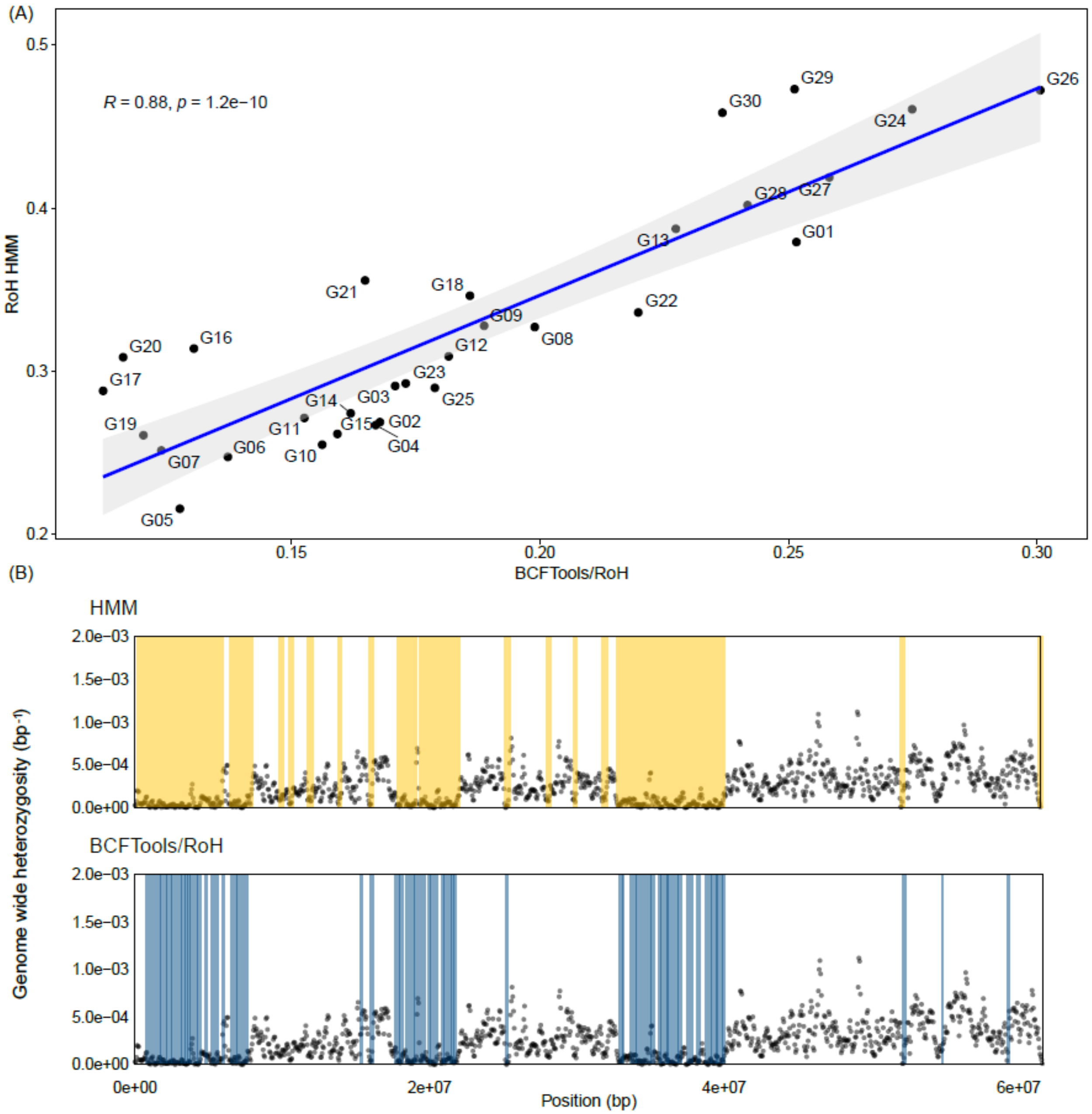


**Figure S9.** (**A**) Correlation between our HMM method and the results from BCFTools/RoH. (**B**) Example of HiC_scaffold_19 from gazelle G09 to show how RoH estimated using BCFTools/RoH (blue) are more fragmented than those obtained using our HMM method (yellow). The y-axis corresponds to the number of heterozygous positions per 150kb window in the scaffold (see Materials and Methods section)

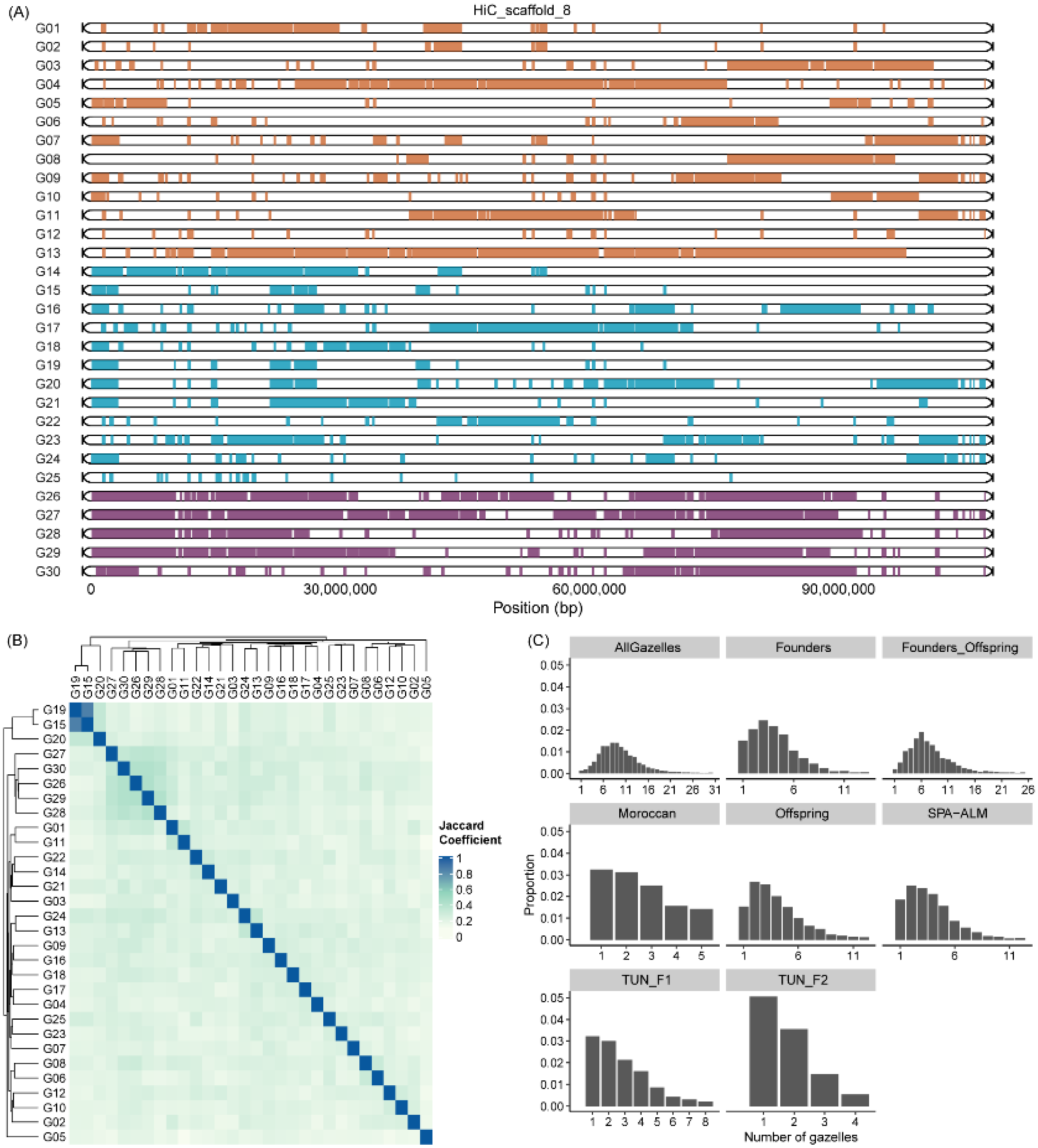


**Figure S10.** (**A**) RoH along HiC_scaffold_8 in all the study gazelles. In orange are the gazelles that belong to the Founders (from G01-G12, SPA-ALM and G13, SPA-CAN), in blue Offspring gazelles (G14-G25) and in purple the Moroccan gazelles (G26-G30). (**B**) RoH similarity matrix using Jaccard coefficients, where 1 represents the highest similarity and 0 no similarity. (**C**) Proportion of RoHs shared by a different number of individuals in different groups of gazelles.

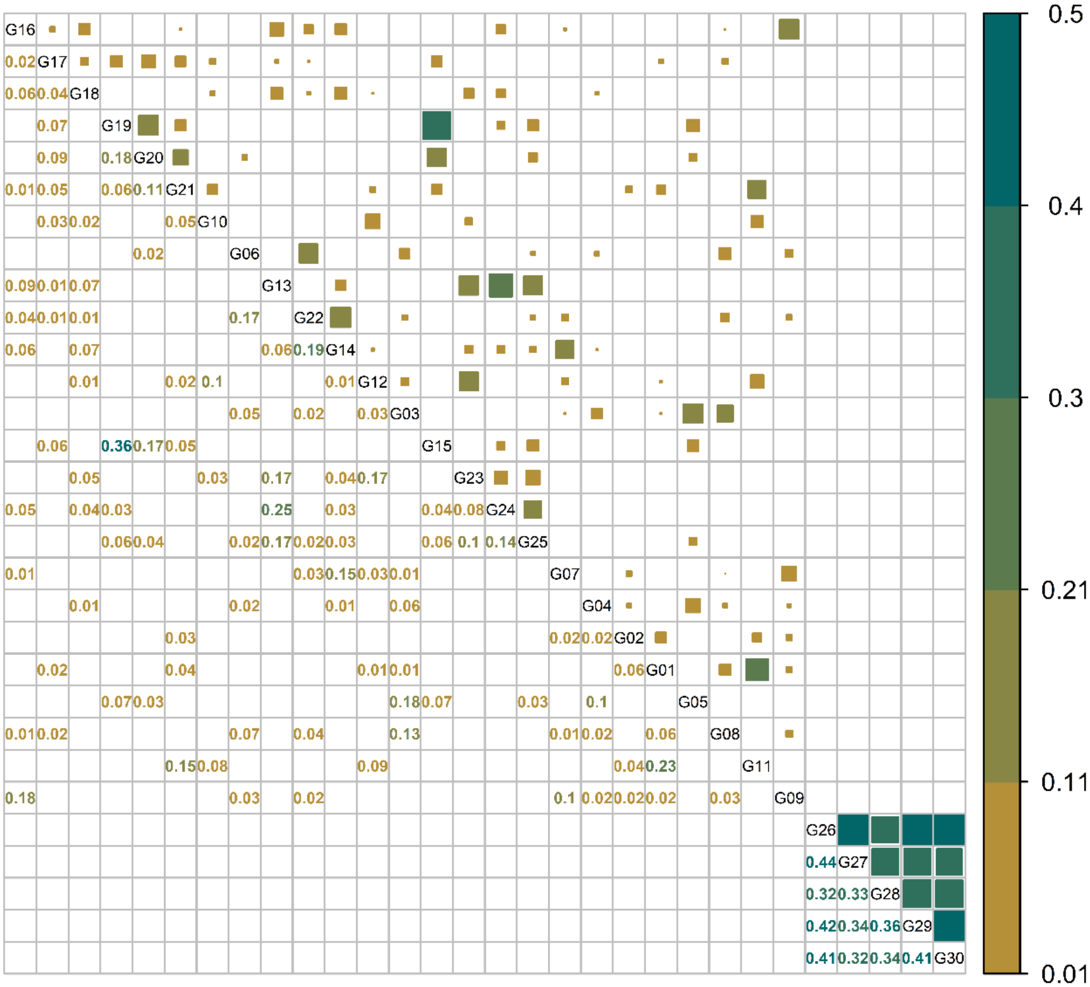

**Figure S11.** NgsRelate V2 results of Founder, Offspring and Moroccan gazelles. The relationship level is shown by the color and the size of the squares (the darker and bigger, the closer the familial relationship). Values indicate the kinship coefficient estimated by the program.

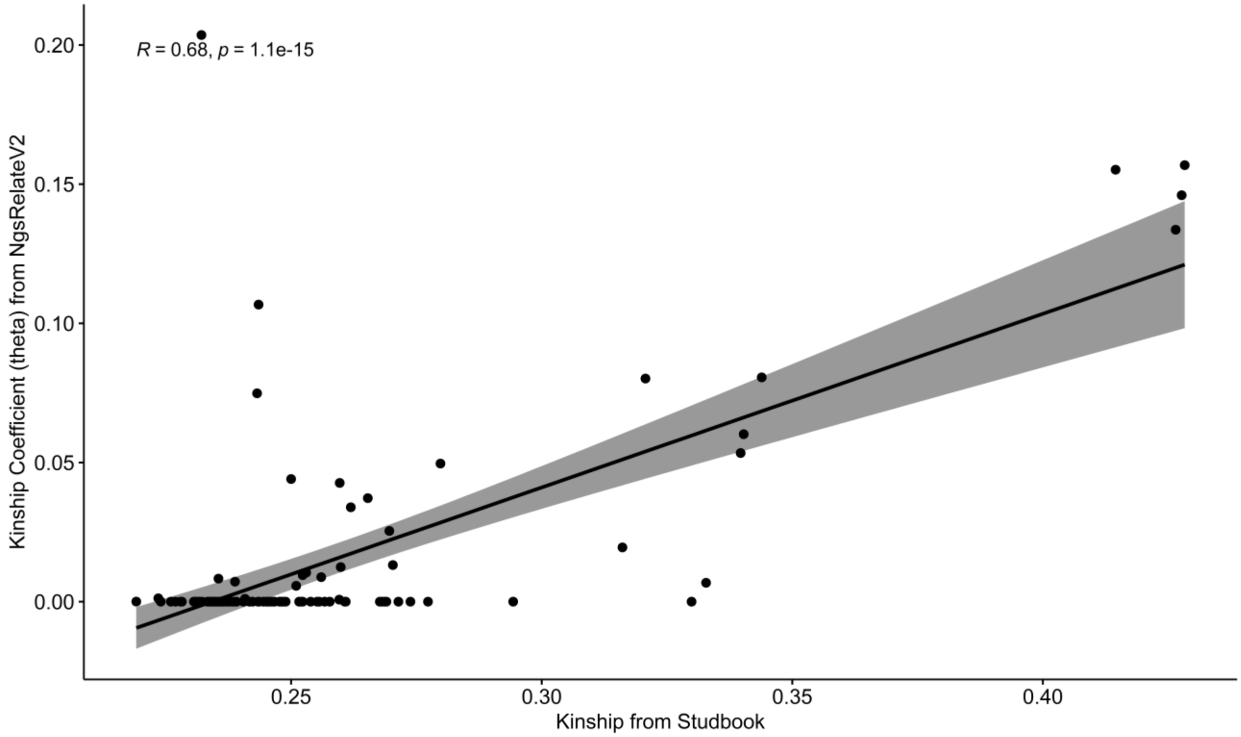


**Figure S12**. Correlation between kinship coefficient estimated using NgsRelateV2 and the kinship coefficient obtained from the Studbook of Cuvier's gazelles (CITATION) using the SPARKs and PMx software.

## *References*

Espeso, G., Moreno, E., 2019. International Cuvier's Gazelle Studbook [WWW Document]. URL http://www.eeza.csic.es/documentos/Studbook_G%20cuvieri%202019.txt (accessed 7.29.20).
